# *Notes from the Field:* Clinical and Epidemiologic Characteristics of Mpox Cases from the Initial Phase of the Outbreak — New York City, May 19–July 15, 2022

**DOI:** 10.15585/mmwr.mm715152a3

**Published:** 2022-12-30

**Authors:** Nang Thu Thu Kyaw, Naama Kipperman, Karen A. Alroy, Jennifer Baumgartner, Addie Crawley, Eric Peterson, Amara Ross, Randal C. Fowler, Victoria E. Ruiz, Mindy Leelawong, Scott Hughes, Mirline Juste-Tranquille, Kevin Lovingood, Celia Deane Joe, Michele Chase, Amanda Shinall, Joel Ackelsberg, Camille Bergeron-Parent, Brittan Badenhop, Sally Slavinski, Vasudha Reddy, Ellen H. Lee

**Affiliations:** ^1^New York City Department of Health and Mental Hygiene, New York, New York; ^2^Epidemic Intelligence Service, CDC.

*Monkeypox virus* (MPXV), an *Orthopoxvirus* that can cause monkeypox (mpox) disease in humans, was rarely seen outside Africa before 2022. Since May 2022, mpox has been reported in multiple countries and regions without endemic transmission, including the United States (*1*). New York City (NYC) quickly became one of the major foci of the 2022 outbreak after the first case in a NYC resident was diagnosed on May 19.* Epidemiologic profiles and clinical characteristics of mpox cases in the United States during this outbreak have been described (*2*,*3*), but previous summaries were limited by incomplete data or inclusion of only a subset of cases (*2*,*3*). Most case investigation data from mpox cases reported to the NYC Department of Health and Mental Hygiene (DOHMH) surveillance system have a high degree of completeness for gender, race or ethnicity, sexual orientation, and clinical signs and symptoms. To describe the characteristics of mpox in NYC, case investigation data for NYC residents with mpox diagnosed during May 19–July 15, 2022, were analyzed. Using a standardized form, DOHMH staff members attempted to interview all NYC residents with probable (a positive non-variola Orthopoxvirus polymerase chain reaction [PCR] test result)^†^ or confirmed (a positive MPXV–specific PCR test result) mpox reported to DOHMH through mandated laboratory reporting. For patients who declined an interview or were unreachable, information obtained from medical care providers during DOHMH consultation calls was used. This activity was reviewed by CDC and conducted consistent with applicable federal law and CDC policy.^§^

Among 719 NYC patients with probable or confirmed mpox, 704 (97.9%) were men; 566 (78.7%) were gay, lesbian, or queer; and 40 (5.6%) were bisexual (Table). Among 651 patients with available data on intimate or sexual exposure, 505 (77.6%) reported intimate or sexual contact with men, and among 611 patients with known contact data, 103 (16.9%) reported contact with persons with suspected mpox during the 3 weeks preceding their symptom onset. Prodromal symptoms were reported by 234 (38.1%) of 614 patients with symptom data; 277 patients (45.1%) reported proctitis or rectal symptoms (e.g., constipation, tenesmus, rectal pain, rectal bleeding, or blood in stool), 192 (69.3%) of whom did not observe perianal skin lesions, and eight (3.0%) of whom did not have any skin lesions when rectal symptoms began. Among 584 patients reporting skin lesions, 216 (37.0%) and 117 (20.0%) had genital and perianal involvement, respectively. Ophthalmic manifestations^¶^ were reported by 38 (6.2%) patients. The median interval from symptom onset to diagnosis was 5 days (range = 3–7 days); 101 (14.0%) patients received tecovirimat, and 35 (4.9%) were hospitalized.

**TABLE:** Characteristics of patients with probable and confirmed mpox (N = 719) — New York City, May 19–July 15, 2022

Characteristic (no. with available information)*	No. (%)
**Age, yrs, median (IQR)**	35 (31–41)
**Gender^†^**	
Female	2 (0.3)
Male	704 (97.9)
Transgender, nonbinary, or genderqueer	12 (1.7)
Unknown	1 (0.1)
**Sexual orientation**	
Bisexual	40 (5.6)
Gay, lesbian, or queer	566 (78.7)
Straight or heterosexual	18 (2.5)
Unknown	95 (13.2)
**Race and ethnicity**	
Asian or Pacific Islander	37 (5.2)
Black or African American	148 (20.6)
Hispanic or Latino	212 (29.5)
White	247 (34.4)
Unknown	75 (10.4)
**Possible exposure ≤3 weeks before symptom onset**	
**Had intimate or sexual contact (n = 651)^§^**	**521 (80.0)**
No. of partners, median (IQR)	3 (1–5)
Contact with men	505 (77.6)
No. of partners, median (IQR)	3 (1–5)
Contact with women	17 (2.6)
No. of partners, median (IQR)	1 (1–2)
Contact with persons who identify as transgender, nonbinary, genderqueer, or other gender identity	7 (1.1)
No. of partners, median (IQR)	1.5 (1–5)
Contact with persons with unknown gender identities	6 (0.9)
No. of partners, median (IQR)	1 (1–3)
Self-reported contact with a person with suspected mpox (n = 611)^¶,^**	**103 (16.9)**
Intimate or sexual contact	66 (64.1)
Household contact	5 (4.8)
Other	13 (12.6)
Unknown	20 (19.4)
**Symptomatic**	
Yes	614 (85.4)
Unknown	105 (14.6)
**Presence of prodrome (n = 614)** ^††^	**234 (38.1)**
**Sign or symptom (n = 614)** ^§§^	
Fever	360 (58.6)
Body or muscle ache, myalgia, or back pain	327 (53.3)
Fatigue	319 (51.9)
Chills	299 (48.7)
Lymphadenopathy	298 (48.5)
Itching or pruritus	291 (47.4)
Proctitis or rectal sign or symptom^¶¶^	277 (45.1)
Proctitis	111 (40.0)
Constipation	100 (36.1)
Tenesmus	102 (36.8)
Rectal pain	211 (76.2)
Rectal bleeding	108 (39.0)
Blood in stool	97 (35.0)
Headache	253 (41.2)
Night sweats	253 (41.2)
Malaise	236 (38.4)
Sore throat	173 (28.2)
Runny nose or cough	118 (19.2)
Gastrointestinal***	96 (15.6)
Ophthalmic manifestations^†††^	38 (6.2)
**Presence of skin lesion (n = 614)**	
Yes	584 (95.1)
Unknown	30 (4.9)
**No. of skin lesions (n = 584)**	
1–9	227 (38.9)
10–49	192 (32.8)
50–99	20 (3.4)
≥100	4 (0.7)
Unknown	141 (24.1)
**Location of skin lesion (n = 584)^§§§^**	
Upper or lower extremities	234 (40.1)
Genitals	216 (37.0)
Face, mouth, or lip	194 (33.2)
Torso (i.e., chest, abdomen, back, or trunk)	184 (31.5)
Perianal	117 (20.0)
Hand or foot	116 (19.9)
Buttocks	108 (18.5)
Scalp, head, or neck	108 (18.4)
Palms or soles	81 (13.9)
Eye	3 (0.5)
Other	85 (14.5)
**Location where skin lesion began (n = 584)**	
Genitals	178 (30.5)
Upper or lower extremities	134 (22.9)
Face, mouth, or lip	98 (16.8)
Torso or back	76 (13.0)
Perianal	72 (12.3)
Palms or soles	42 (7.2)
Neck	28 (4.8)
Other	87 (14.9)
Unknown	143 (24.5)
**HIV infection, self- or provider-reported**	181 (25.2)
**Interval from symptom onset to diagnosis, days, median (IQR)**	5 (3–7)
**Receipt of PEP with JYNNEOS vaccine 0–14 days after last exposure**	10 (1.4)
**Initiated treatment with tecovirimat**	101 (14.0)
**Hospitalized **	35 (4.9)

**Abbreviations: **mpox = monkeypox; PEP = postexposure prophylaxis.

* Unknown category included patients with missing data or patients who responded “do not know” or “declined to answer” to the survey question.

^†^ Transgender women or men are included with transgender, nonbinary, or genderqueer. Groups are mutually exclusive.

^§^ Patients could report intimate or sexual contact with more than one gender.

^¶^ Suspected mpox case defined as a person with a diagnosis of mpox or with compatible signs or symptoms. Patient could report more than one type of contact.

** Percentage of persons who reported contact with a person with suspected mpox.

^††^ Presence of nondermatologic signs or symptoms before onset of skin lesion.

^§§^ Patients could have more than one sign or symptom.

^¶¶^ Percentage of patients with proctitis or rectal signs or symptoms.

*** Includes nausea, vomiting, abdominal pain, or discomfort.

^†††^ Includes eye lesion, conjunctivitis, red eyes, or eye discharge.

^§§§^ Patient could have skin lesions in more than one location.

Data on gender, race or ethnicity, and sexual orientation from the DOHMH surveillance system were >80% complete for patients with mpox diagnosed during the study period; these data informed public health outreach and intervention efforts.** Whereas ophthalmic involvement has been rarely reported in the current global mpox outbreak, 38 (6.2%) of 614 patients in this analysis reported ophthalmic manifestations, which can require urgent clinical management and result in longer-term sequelae (*4*). More than two thirds of patients with proctitis or rectal symptoms did not report perianal skin lesions. Moreover, a small number of these patients reported no skin lesions at onset of rectal symptoms. Although the current recommended method of swabbing skin lesions to diagnose mpox does not include collection of anorectal swabs, MPXV can be detected from anorectal swabs in patients with rectal symptoms^††^ (*5*). Additional studies evaluating anorectal swabs for use in mpox diagnosis might expand the range of potential specimens and enhance the possibility for early diagnosis among symptomatic persons at risk for mpox but without cutaneous lesions.

The findings in this report are subject to at least two limitations. First, data were missing for approximately 15% of patients, more than one half of whom were unreachable for interview; these persons might differ systematically from those who were interviewed. Second, mpox testing and treatment resources were limited during the study period, and the epidemiology has since evolved; thus, these findings might not be generalizable throughout the outbreak.

These findings can guide development of public health messaging to communities with increased likelihood of exposure to mpox; in addition to avoiding close personal contact with someone with mpox and recommendations for eligible persons to receive mpox vaccine, the clinical manifestations described in this report can guide providers managing patients with mpox. Further studies are needed to assess the potential utility of anorectal swabs in early diagnosis of mpox.

## Footnotes

*
https://www.nyc.gov/site/doh/data/health-tools/monkeypox.page

† Swabs from skin lesions were tested for *Orthopoxvirus* or non-variola *Orthopoxvirus* at DOHMH Public Health Laboratory, commercial, or academic laboratories. CDC tested swabs submitted from DOHMH for *Orthopoxvirus* or non-variola *Orthopoxvirus* in addition to an MPXV-specific test.

§ 45 C.F.R. part 46.102(l)(2), 21 C.F.R. part 56; 42 U.S.C. Sect. 241(d); 5 U.S.C. Sect. 552a; 44 U.S.C. Sect. 3501 et seq.

¶ Includes eye lesion, conjunctivitis, red eyes, or eye discharge.

**
https://www.nyc.gov/site/doh/about/press/pr2022/health-department-releases-monkeypox-vaccination-demographic-data.page

††
https://www.cdc.gov/poxvirus/monkeypox/about/science-behind-transmission.html
